# BioGoldNCDB: A Database of Gold Nanoclusters and Related Nanoparticles with Biomedical Activity

**DOI:** 10.3390/molecules30153310

**Published:** 2025-08-07

**Authors:** Eszter Erdei, András Mándoki, Andrea Deák, Balázs Balogh, László Molnár, István M. Mándity

**Affiliations:** 1 Institute of Organic Chemistry, Semmelweis University, Hőgyes Endre u. 7, H-1092 Budapest, Hungary; erdei.eszter@ttk.hu (E.E.); mandoki.andras@ttk.hu (A.M.); balogh.balazs@semmelweis.hu (B.B.); 2 Artificial Transporter Research Group, Institute of Materials and Environmental Chemistry, HUN-REN Research Centre for Natural Sciences, Magyar Tudósok krt. 2, H-1117 Budapest, Hungary; molnar.laszlo@ttk.hu; 3 Center for Pharmacology and Drug Research & Development, Semmelweis University, H-1085 Budapest, Hungary; 4 Solid Science Ltd., Podmaniczky utca 57, H-1064 Budapest, Hungary

**Keywords:** gold, nanocluster, database, application, particle size, therapy

## Abstract

Interest in gold nanoclusters (AuNCs) has grown significantly in recent decades. AuNCs, with a core size smaller than 2 nm, represent a unique class of functional nanomaterials. Their distinctive properties enable innovative applications across various interdisciplinary fields. Here, we introduce BioGoldNCDB, a freely available, fully annotated, and manually curated database of mainly about AuNCs and related AuNPs. Despite the rapid growth in biomedical applications of gold nanoclusters (AuNCs), the lack of a centralized and structured data resource hinders comparative analysis and rational design. Researchers face challenges in accessing standardized information on AuNCs’ structures, properties, and biological activities, which limits data-driven development in this emerging field. The database provides essential information, including CAS numbers and PubMed IDs, as well as specific details such as biomedical applications, cell lines used in research, particle size, and excitation/emission wavelengths. It currently covers 247 articles from 104 journals. Designed with a user-friendly and intuitive web interface, BioGoldNCDB is accessible on multiple devices, including phones, tablets, and PCs. Users can refine searches with multiple filters, and a help page is available for guidance. While offering quick insights for newcomers, BioGoldNCDB also serves as a valuable resource for researchers across various fields.

## 1. Introduction

Bionanotechnology has been a rapidly advancing field in recent decades, integrating chemistry, physics, biology, and medicine with nanotechnology to drive innovation in modern science [1,2,3]. Metallic nanoparticles (NPs) play a crucial role in this field due to their unique properties. They can be synthesized and functionalized with various groups, enabling conjugation with peptides, antibodies, ligands, or even drugs [4]. This versatility opens up a wide range of biomedical applications, including diagnostic imaging, biosensing, sample preparation, and therapy [5,6].

NPs typically range in size from 1 to 150 nm [1,7] and exhibit size-dependent applications and distinctive physical properties, allowing them to interact with cell surface receptors and biomolecules. Additionally, they serve as effective drug delivery agents [8]. Among metallic NPs, a few materials stand out, particularly iron oxide NPs (Fe_3_O_4_, Fe_2_O_3_) [7,9,10,11], which possess magnetic properties, and silver NPs, which have gained attention due to their antimicrobial and biomedical applications [12,13,14]. These materials are widely utilized in protein immobilization, MRI imaging, targeted drug delivery, infectious disease treatment, and inflammation control [7,15,16].

AuNPs are among the most widely explored nanomaterials due to their exceptional optical properties, stability, and surface functionalization potential. Their surface can be modified for bioconjugation with peptides, proteins, oligonucleotides, and antibodies [17,18]. AuNPs can function as artificial antibodies, with their binding affinity adjustable by altering the ligand density on their surface [19]. Their multivalency allows them to protect unstable drugs or poorly soluble contrast agents, facilitating effective delivery to otherwise inaccessible tissues. Due to their unique nanoscale properties, AuNPs can modulate cellular processes in ways that small molecules or proteins cannot [20]. Some gold NPs efficiently convert light into heat, enabling precise thermal ablation of diseased tissues. Additionally, their high X-ray absorption enhances cancer radiation therapy and improves imaging contrast in computed tomography (CT) scans. Metallic NPs, in general, exhibit strong absorption and vivid colors due to surface plasmon resonance (SPR) [21], but their fluorescence [22] or luminescence [23] emission is generally weak or negligible. However, gold nanotechnology enables the integration of multiple functionalities into a single construct, allowing for simultaneous targeting, diagnosis, and therapy [24]. These properties make AuNPs an attractive platform for personalized medicine, as they can be chemically tailored for specific diseases or patients [24,25,26,27,28,29,30,31].

**Scheme 1:**
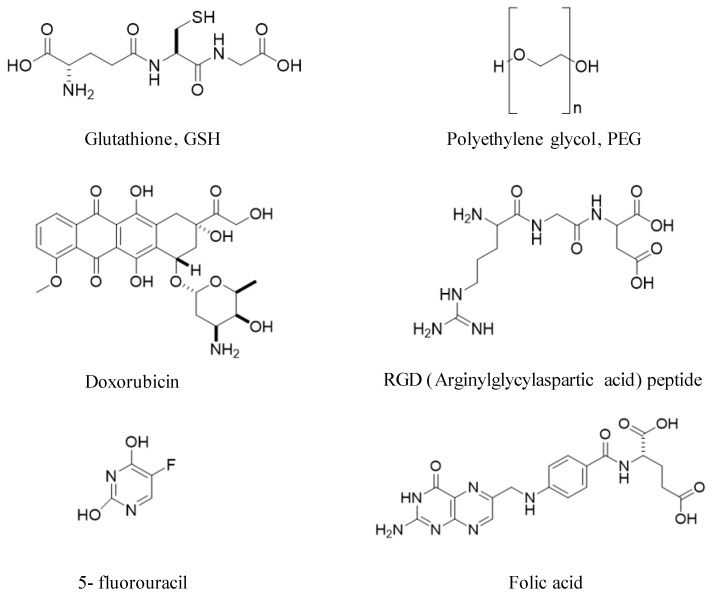
Structures of the most common nanoconjugates and ligands.

Gold nanoclusters (AuNCs) are atomically precise gold nanomaterials typically smaller than 2 nm, exhibiting discrete electronic structures and molecular-like properties. These features fundamentally distinguish them from larger gold nanoparticles (AuNPs), which display bulk-like and plasmonic behavior. Therefore, AuNCs are more accurately regarded as a separate class of nanomaterials, rather than a subclass of AuNPs. Unlike standard AuNPs, AuNCs [32] significantly alter their optical, electronic, and catalytic behaviors. Solid-phase techniques [33] have become more prevalent due to their ease of use and scalability. AuNCs are typically synthesized in the presence of specific ligands, which act as stabilizing agents by forming strong interactions with metal atoms (Scheme 1). Thiolated ligands [34], in particular, directly interact with the Au core via weak coordination bonds, enhancing their sensing and catalytic properties.

Due to their ultrasmall size, AuNCs display molecule-like behavior rather than traditional NP characteristics. This is because their dimensions are comparable to the Fermi wavelength [35] of electrons, leading to the disappearance of conventional metallic properties. Instead, pronounced quantum confinement effects cause the energy bands to transform into discrete electronic states, resulting in distinct optical properties compared to larger NPs [36]. These clusters (Au_10_, Au_25_, Au_38_, Au_144_) are part of the “magic-number” series of thiolate-protected gold superatoms exhibiting enhanced stability due to closed electronic shells [37,38,39,40].

The biocompatibility, photostability, and unique optical properties of AuNCs make them promising candidates for various biomedical applications [28,41,42]: (*i*) fluorescent AuNCs serve as bioimaging and cell imaging agents due to their non-toxic core [43]; (*ii*) functionalized AuNCs exhibit enzyme-mimicking activity, enabling their use in protein activity inhibition and colorimetric detection of analytes [28,44,45]; and (*iii*) ultrasmall AuNCs demonstrate broad-spectrum antimicrobial properties, efficiently targeting both Gram-positive and Gram-negative bacteria by disrupting bacterial metabolism and inducing intracellular reactive oxygen species (ROS) [46] accumulation, ultimately leading to bacterial cell death [47,48,49,50,51].

Over the past few years, a large number of reports have been published to demonstrate applications of AuNCs in oncological cases, ranging from imaging [52] and diagnostic [53] to targeted therapy [54,55], radiotherapy [56,57], and immunotherapy [58]. Their efficacy has been extensively tested in various cancer cell lines, such as HeLa [59], HepG2 [60], A549 lung cancer cells [61], MCF-7 [62], U87 [63] and MDA-MB-231 [64], and even in mice models [65].

A database was created, namely BioGoldNCDB (Bio = biomedical, NC = nanocluster, DB = database), where all the necessary information about gold nanoclusters and related nanoparticles can be found. The database is accessible at https://biogoldncdb.ladon.life/ accessed on 15 August 2024.

## 2. Results

### Analysis of Conjugate Applications

**Figure 1:**
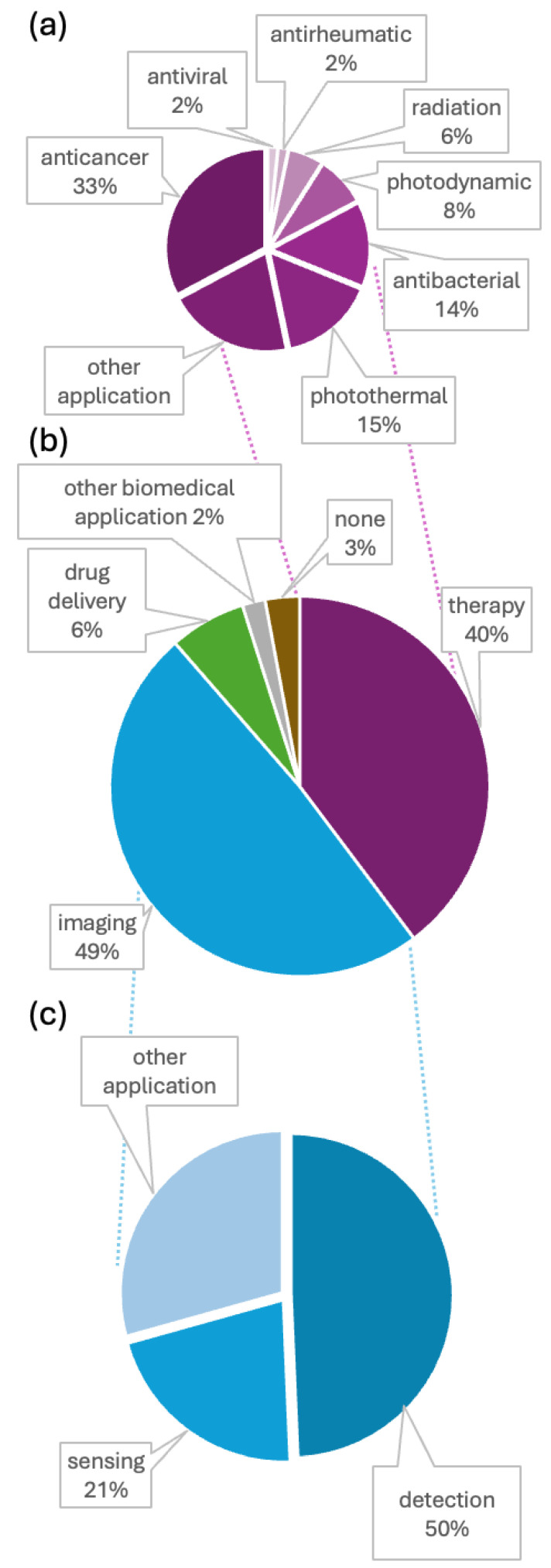
The distribution of applications found for the BioGoldNCDB. (**b**) A more specific separation was made for therapy (**a**) and imaging (**c**).

The biomedical applications included in BioGoldNCDB and their distribution are shown in Figure 1b. The most common applications are therapy (122 entries) and imaging (150 entries).

**Figure 2:**
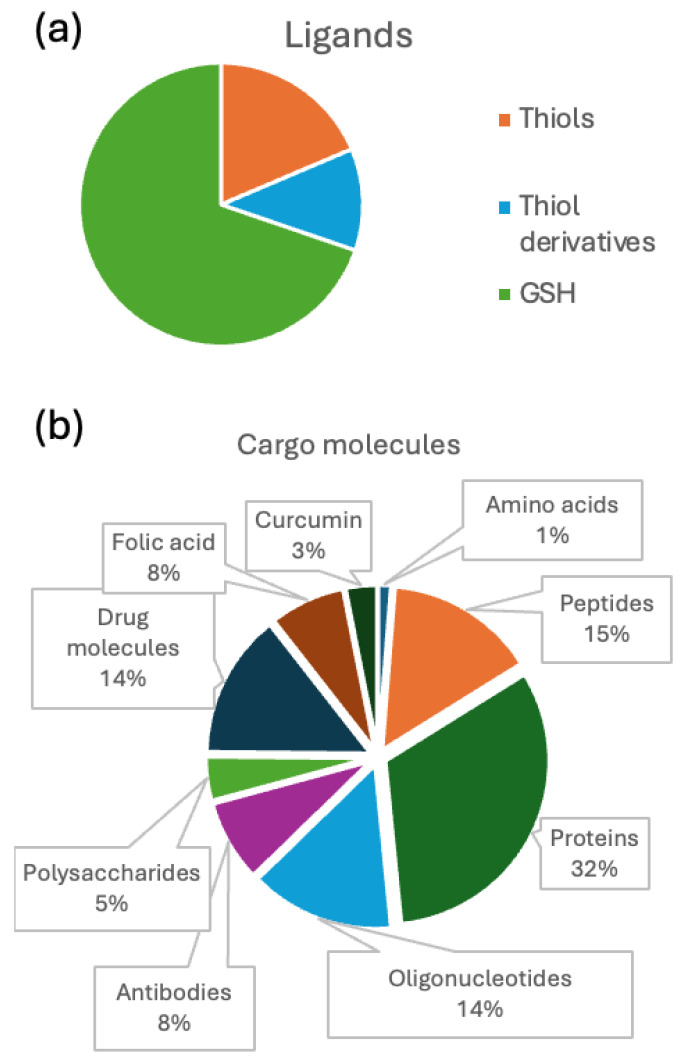
The distribution of ligands (**a**) and cargos (**b**) found for the BioGoldNCDB entries.

The most relevant nanoconjugates and their distribution in BioGoldNCDB are presented in Figure 2. The surface ligands of AuNCs play a critical role in determining their stability, photophysical properties and interactions with additional biomolecules. Thiols and thiol derivatives are the primary functionalization agents for AuNCs (Figure 2a). Glutathione (GSH) is the most commonly used thiolated ligand for the stabilization and functionalization of AuNCs. The covalent or noncovalent conjugation of additional biomolecules or other cargo molecules to AuNCs enables the formation of nanobioconjugates, enhancing their functionality, targeting, and activity in biological environments. Among cargo molecules, peptides and proteins are the most frequently utilized, though oligonucleotides and drug molecules are also common (Figure 2b).

**Figure 3:**
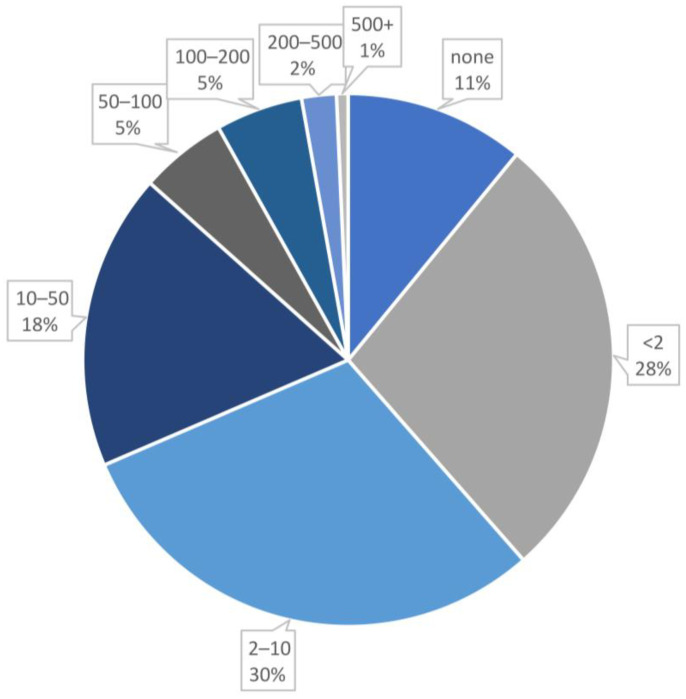
The distribution of the complete particle sizes found for the BioGoldNCDB entries given in nm.

The size distribution of AuNCs is shown in Figure 3. During data collection, our primary goal was to obtain the gold core size; however, in many cases, only size data for the nanobioconjugates were available, or the original core size was not determined.

In the case of AuNCs, 28% are smaller than 2 nanometers, while related nanoparticles make up nearly half of the cases (specifically, 30% are between 2 and 10 nm, and 18% are between 10 and 50 nm).

**Figure 4:**
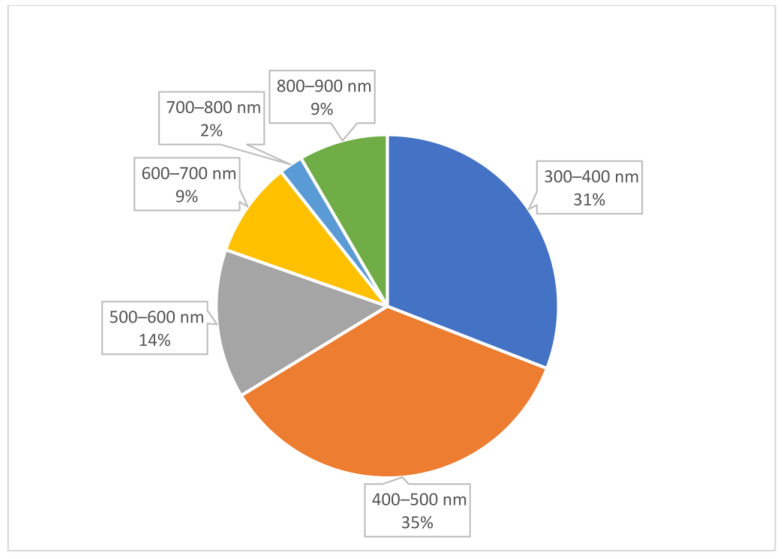
The distribution of excitation wavelengths found for the BioGoldNCDB entries.

**Figure 5:**
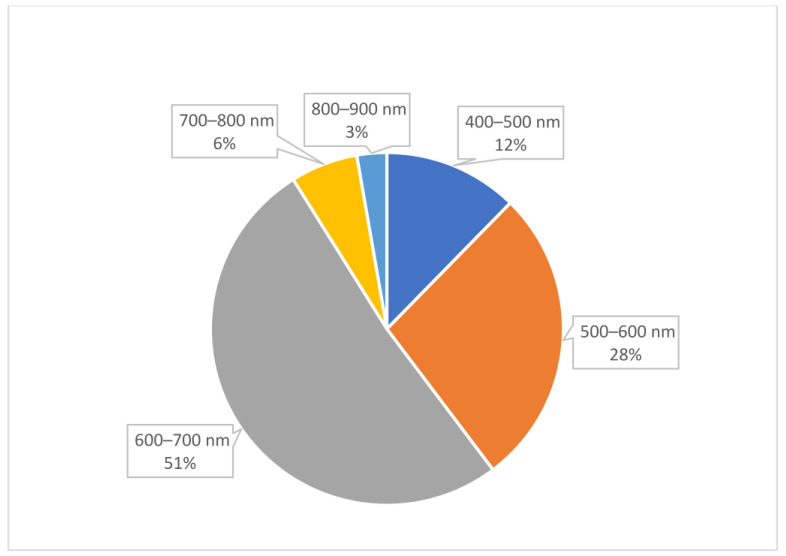
The distribution of emission wavelengths found for the BioGoldNCDB entries.

Excitation and emission wavelength data were collected from the articles and are presented separately in Figure 4 and Figure 5. Most excitation wavelengths fall within the 400–500 nm range, while emission wavelengths are primarily in the 600–700 nm range. Notably, excitation wavelength data were missing in 33% of cases, and emission wavelength information was absent in 39% of cases.

In several cases, gold NCs are combined with one or more additional metals, forming hybrid structures. These hybrids were excluded from our analysis, as the conjugated metals alter the properties of gold, thereby affecting the statistical outcomes.

**Figure 6:**
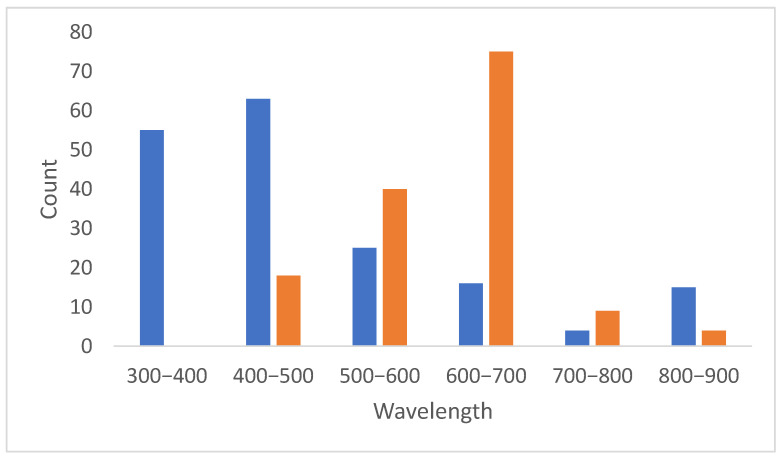
Distribution of excitation (blue) and emission (orange) wavelengths for gold nanoclusters (AuNCs), grouped in 100 nm intervals. The majority of AuNCs exhibit excitation in the lower wavelength range (300–500 nm), while their emission typically occurs at higher wavelengths (600–700 nm), reflecting their characteristic photophysical behavior.

The number of AuNCs with reported excitation and emission wavelengths, categorized into 100 nm intervals, is shown in Figure 6. These data clearly demonstrate that most AuNCs are excited at lower wavelengths (300–500 nm), while their emission predominantly occurs at higher wavelengths, typically in the 600–700 nm range.

## 3. Discussion

### 3.1. Database Overview

#### 3.1.1. Database Content and User Interface Layout

The BioGoldNCDB search function enables users to efficiently explore the database using multiple search criteria. It contains information on gold NCs compiled from 247 published research articles. Users can search by keywords related to NC characteristics, particle size, and biomedical applications. Advanced filters allow for refinement based on particle size, surface modification (nanobioconjugates), and other key parameters. Additionally, BioGoldNCDB integrates SciFinder’s data to enhance searches with valuable extra information. Users can further narrow results by specifying CAS numbers, journal names, or other identifiers. By combining these search options, researchers can quickly access specific and relevant data, facilitating the discovery and application of gold NPs in drug delivery, imaging, and diagnostics. The database serves as a valuable resource for both experienced researchers and those new to the field. Its content is categorized into four main groups for easy navigation. BioGoldNCDB features an intuitive and highly responsive user interface, enabling effortless data exploration with column sorting, filtering, and inline editing. With built-in support for keyboard navigation, drag-and-drop functionality, and export options, it enhances usability, allowing users to efficiently manage and interact with large datasets.

#### 3.1.2. Manually Curated Data

- **Application:** The biomedical field in which the AuNCs can be utilized.
- **Cell line:** The specific cell culture used for testing or biomedical investigation.
- **Particle size:** Includes both the core size (analyzed by TEM technique) and the total size of the nanobioconjugate, measured in nanometers (nm).
- **Excitation/emission wavelength:** The **excitation wavelength** refers to the specific wavelength of light that is absorbed by gold AuNCs to promote their electrons to an excited energy state. The **emission wavelength** is the wavelength of light emitted as the excited electrons return to their ground state.
- **Nanobioconjugates:** Any molecule or material added to gold NCs as part of the functionalization process.
- **TOC:** Table of Contents figures.

#### 3.1.3. Bibliographic Information

- **ID:** A unique identifier assigned to the article by the database or journal where it is published. (Usually not shown in the article itself).
- **Title:** The main heading of the article that concisely captures its content.
- **Abstract:** A brief summary of the article’s purpose, methodology, key findings, and conclusions.
- **Author (s):** The person (people) who conducted the research and wrote the article.
- **Journal:** The scientific publication where the article appears.
- **Organization:** The affiliation of the author (s), typically their university or research institution.
- **Database:** The online platform where the article can be accessed (e.g., PubMed Central, ScienceDirect).
- **Volume:** The volume number of the journal issue where the article is published.
- **Issue:** The specific issue number within the volume.
- **Paper:** (uncommon) Sometimes used interchangeably with article.
- **Page numbers:** The page range where the article appears within the journal issue.
- **Publication date:** The date the article was published in the journal.

#### 3.1.4. Identification

- **Concepts:** The main topics or ideas explored in the article.
- **CAS Numbers:** Unique identifiers assigned by the Chemical Abstracts Service to chemical substances mentioned in the article.
- **Keywords:** A list of terms relevant to the article’s subject matter used for indexing and searchability.
- **Accession Number:** An identification number assigned to the article by a specific database.
- **Chemical Abstracts Number (CAN):** Unique identifier for the abstract text of the publication assigned by Chemical Abstracts Service.
- **Section:** The specific section of the journal where the article is published (e.g., research articles, reviews).
- **Section Cross-Reference:** A reference to a related section within the journal where additional relevant information might be found.

#### 3.1.5. Identifiers

- **CODEN:** A unique six-character code assigned to a specific journal title.
- **DOI (Digital Object Identifier):** A unique identifier for an article that remains constant even if the location of the article changes online.
- **Journal Code:** An abbreviation used to identify a specific journal.
- **PubMed ID:** A unique identifier assigned to an article by the PubMed database.
- **MEDLINE MeSH (Medical Subject Headings):** A controlled vocabulary used by MEDLINE/PubMed to index articles in the life sciences.

### 3.2. Examples

**Figure 7:**
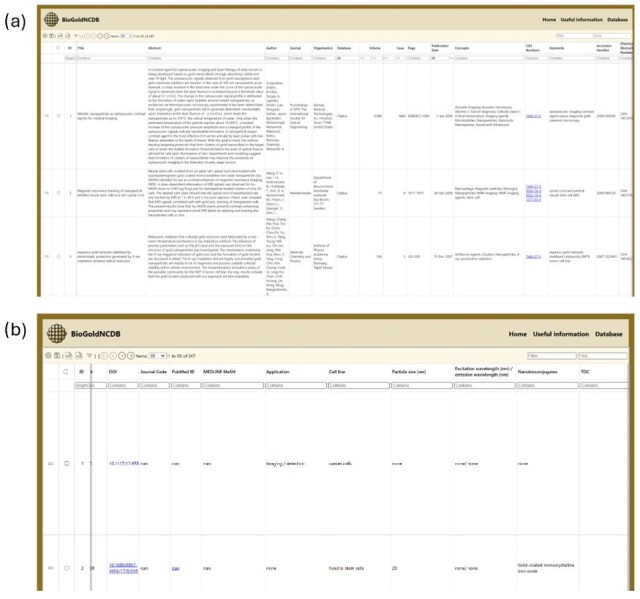
The layout of BioGoldNCDB, displaying database entries melyek (**a**) and amelyek (**b**), with columns accompanied by various search options.

Below are brief examples of some search options available in BioGoldNCDB (Figure 7):

1. **Free Text Search:** A keyword-based free text search enables users to explore various database fields, including BioGoldNCDB ID, CAS number, PubMed ID, author name, article title, organization, journal name, and year of publication. More details are available at https://biogoldncdb.ladon.life/.
2. **Simple Search:** Users can also search for specific entries in BioGoldNCDB based on application, cell line type, particle size, nanoconjugates, and excitation/emission wavelengths, etc.
3. **Multi-criteria search:** Users can also search using multiple criteria in BioGoldNCDB.

**Figure 8:**
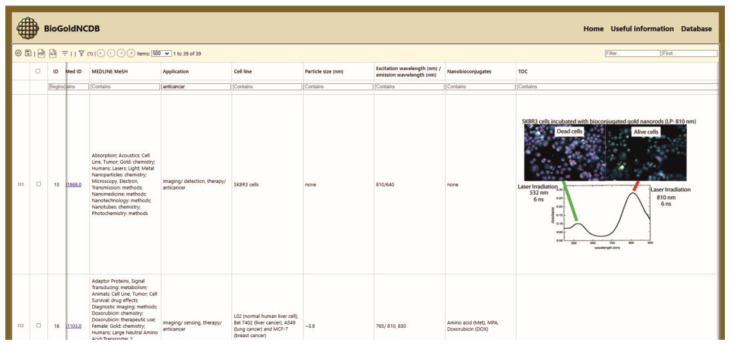
An illustration of a BioGoldNCDB for single search example.

The platform offers several user-friendly features. Users can filter data by specific columns (e.g., searching for the keyword ‘anticancer’ within the application column) or perform a comprehensive keyword search across the entire database. Additionally, results can be exported in PDF and Excel formats using dedicated buttons, and applied filters can be saved within the database. As an example of a single search, searching for ‘anticancer’ returns 39 results for gold NCs with anticancer properties (Figure 8).

**Figure 9:**
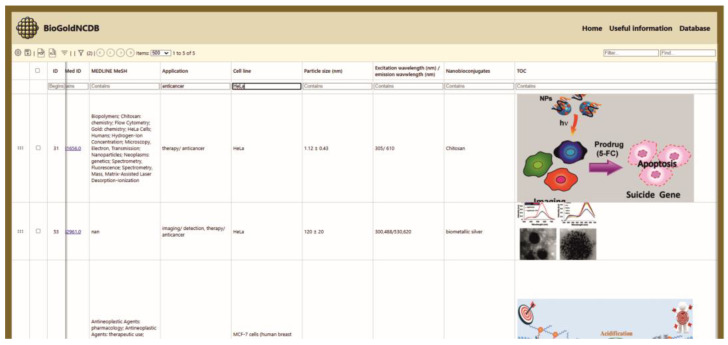
An illustration of a BioGoldNCDB for multi-criteria search example.

The database allows for multi-criteria searches using two or more keywords. For example, instead of searching only for anticancer applications, we can also filter for entries tested on the HeLa cell line. Using these criteria, only five results were found (Figure 9).

## 4. Materials and Methods

### 4.1. Data Collection and Processing

Each entry in BioGoldNCDB is a manually curated and annotated record of gold NCs collected from the literature. The initial search was performed using the keyword ‘gold nanocluster’ in the Chemical Abstracts Service (CAS) database (https://www.cas.org/) via SciFinder (https://scifinder-n.cas.org/). Search filters were applied to include publications from 2000 to 2024, journal articles (as document type) in English, and substances with roles in diagnostic use and pharmacological activity were included only. Review articles were excluded, resulting in 3577 articles. A final filter retained only those whose abstracts contained the keyword ‘cluster’, refining the selection further. Six additional manually curated data fields were incorporated, covering application, cell line, particle size, excitation/emission wavelength, nanobioconjugates, and table of content (TOC) graphics. The search was conducted on 27 June 2024. Each literature entry was manually reviewed, and only those specifically related to gold NCs and related nanoparticles were included in the final database. The total number of selected articles was 247.

### 4.2. Database Design and Implementation

The BioGoldNCDB web interface was developed using Next.js and React for the front end [66], while back-end processes are managed within a Node.js environment [67]. Data storage relies on a MySQL relational database, with queries executed through MySQL commands [68]. The spreadsheet-style data visualization on the webpage was built using the EZGrid React DataGrid component [69]. Both the website and database are hosted on DigitalOcean’s cloud infrastructure [70].

## 5. Conclusions

In conclusion, we have developed BioGoldNCDB, a publicly available database that compiles extensive information on AuNCs and related AuNPs, their conjugates, and relevant literature. The database features an intuitive, user-friendly interface, making it accessible not only to experienced researchers but also to newcomers in the field. All data were manually collected and processed to present relevant insights into the biocompatibility, usability, and key properties of gold NCs in this rapidly evolving field. Each entry includes basic details such as the abstract, CAS Number (CAS RN), Chemical Abstracts Number (CAN), DOI, and PubMed ID. Additionally, the database provides information on applications, particle size, excitation and emission wavelengths, investigated cell lines, and nanoconjugates used.

BioGoldNCDB currently contains 247 entries sourced from 104 different journals. To our knowledge, this is the first freely available comprehensive database dedicated to gold NCs. We believe that BioGoldNCDB highlights the significance of this unique class of nanomaterials and showcases their promising biomedical potential.

While the database serves as a valuable resource for newcomers, we are confident that advanced researchers will also find substantial information relevant to this important field.

Future developments for BioGoldNCDB include periodic incorporation of newly published experimental data, potential integration with related nanomaterial databases to enhance interoperability, and the implementation of advanced features such as customizable filtering options, interactive data visualizations, and improved user navigation to support diverse research needs. While our primary focus remains on gold nanoclusters (AuNCs), we have also included related gold nanoparticles (AuNPs) when they were reported in the same original studies, in order to preserve the context and completeness of the biomedical findings.

## Data Availability

All database entries are freely available at https://biogoldncdb.ladon.life/. Accessed on 15 August 2024.

## Abbreviations

The following abbreviations are used in this manuscript:

AuNCsI	Gold nanoclusters
AuNPsJ	Gold nanoparticles
CT	Computed tomography
GSH	Glutathione
SPR	Surface plasmon resonance
